# Anterior Commissure Regulates Neuronal Activity of Amygdalae and Influences Locomotor Activity, Social Interaction and Fear Memory in Mice

**DOI:** 10.3389/fnmol.2020.00047

**Published:** 2020-03-31

**Authors:** Tsan-Ting Hsu, Tzyy-Nan Huang, Yi-Ping Hsueh

**Affiliations:** Institute of Molecular Biology, Academia Sinica, Taipei, Taiwan

**Keywords:** anterior commissure, associative memory, basolateral amygdala, hyper-locomotor activity, social interaction, sucrose preference

## Abstract

The two hemispheres of the vertebrate brain are connected through several commissures. Although the anterior commissure (AC) is the most conserved white matter structure in the brains of different vertebrates, its complete physiological functionality remains elusive. Since the AC is involved in the connection between two amygdalae and because amygdalae are critical for emotional behaviors and social interaction, we assessed amygdalar activity and function to investigate the physiological role of the AC. We first performed *ex vivo* electrophysiological recording on mouse brains to demonstrate that the AC delivers a positive signal to facilitate synaptic responses and to recruit basolateral amygdalar neurons via glutamatergic synapses. Transection was then undertaken to investigate the role of the AC *in vivo*. Results from *in vivo* optogenetic stimulation suggest that AC transection impairs mutual activation between two basolateral amygdalae. Behavioral analyses were then used to assess if AC surgical lesioning results in hyperactivity, anxiety, social reduction or learning/memory impairment, which are behavioral features associated with neuropsychiatric disorders, such as autism spectrum disorders. We found that AC transection results in higher locomotor activity, aberrant social interaction and reduced associative memory, but not anxiety. Moreover, systemic administration of D-cycloserine, a coagonist of N-methyl-D-aspartate receptor, ameliorated auditory fear memory in AC-transected mice, reinforcing our evidence that the AC potentiates the activity of basolateral amygdalae. Our study suggests that the AC regulates basolateral amygdalar activity and influences neuropsychiatry-related behaviors in mice.

## Introduction

Abnormal connectivity between the two brain hemispheres is associated with neurodevelopmental or neuropsychiatric disorders (Geschwind and Levitt, 2007; Valera et al., 2007; Dhar et al., 2010; Xu et al., 2013; Ribolsi et al., 2014). For instance, abnormal development of the anterior commissure (AC) has been found in patients suffering from various neuropsychiatric disorders, including autism (Dodero et al., 2013; Mimura et al., 2019), bipolar disorders (Nadler et al., 2004; Saxena et al., 2012) and schizophrenia (Kikinis et al., 2015). The AC is a highly conserved white matter structure in all vertebrates (Suarez et al., 2014). It contains two major tracts, the anterior and posterior parts in rodents, to link the rostroventral parts of the two brain hemispheres, including the olfactory bulbs, the olfactory tubercles, the anterior piriform cortices, the amygdalae, the perirhinal cortices, and the entorhinal cortices (Horel and Stelzner, 1981; Jouandet and Hartenstein, 1983). Its evolutionary conservation and association with neuropsychiatric disorders suggest a critical role of the AC in brain function.

Based on studies of mouse genetic models and human genetic analyses, many proteins have been shown to regulate AC formation, including guidance molecules, and their receptors (such as epherins/eph receptors Kullander et al., 2001; Robichaux et al., 2016, and semaphorin/plexin receptors Falk et al., 2005; Suto et al., 2005), cell adhesion molecules (Zhou et al., 2008; Abudureyimu et al., 2018; Mimura et al., 2019), cytoskeleton proteins and their regulators (Deuel et al., 2006; Klingler et al., 2015; Dobyns et al., 2018), and transcriptional factors (Huang et al., 2014, 2019b). T-Brain-1 (TBR1), a causative gene of autism spectrum disorders (ASD) (Neale et al., 2012; O'roak et al., 2012a,b; de Rubeis et al., 2014; Sanders et al., 2015), is a brain-specific transcriptional factor involved in AC formation (Huang et al., 2014, 2019b). *Tbr1* is restrictively expressed in projection neurons of the cerebral cortex, olfactory bulbs, amygdalae and hippocampus (Bulfone et al., 1995; Hevner et al., 2001; Huang et al., 2014, 2019b). *Tbr1* haploinsufficiency results in absence or reduction of both anterior and posterior parts of the AC in mice (Huang et al., 2014, 2019b). TBR1 regulates the expression of a set of genes controlling cell adhesion—including *Cntn2, Ntng1*, and *Cdh8*—in the basolateral amygdalae (BLA), thereby mediating axonal projection of BLA neurons to the contralateral side via the AC (Huang et al., 2014; Chuang et al., 2015). Importantly, haploinsufficiency or ASD-linked mutation of *Tbr1* also results in autism-like behaviors in mice, including reduced social interaction, impaired amygdala-dependent associative memory and disrupted olfactory discrimination (Huang et al., 2014, 2019a; Yook et al., 2019), highlighting the relevance of *TBR1* deficiency in ASD and reinforcing the potential role of the AC in neuropsychiatric disorders.

A recent study of common marmosets (*Callitrhix jacchus*), a non-human primate, also showed the alteration of white matter structure by an ASD condition (Mimura et al., 2019). Maternal exposure to valproic acid (VPA) resulted in social deficits of offspring in adulthood, which is a well-known ASD condition induced by environmental factors (Miyazaki et al., 2005; Schneider and Przewlocki, 2005). Marmosets exposed to VPA at fetus stage exhibited downregulation of 20 genes in their brains and presented smaller AC and corpus callosum (CC) (Mimura et al., 2019), suggesting an association of VPA–induced ASD with the altered white matter structure in the brains of these primates. However, in both *Tbr1*^+/−^ mice and VPA-treated marmosets, alterations are not limited to the AC. For instance, the CC is also smaller in the brains of VPA-treated offspring (Mimura et al., 2019), and TBR1 also influences neuronal activity via cell-autonomous regulated expression of N-methyl-D-aspartate receptor subunit 2B (NMDAR2b) (Wang et al., 2004b; Huang et al., 2014).

A previous study applied AC transection to demonstrate that the AC is required for information transfer between the two olfactory bulbs and rapid localization of an odor source (Esquivelzeta Rabell et al., 2017). Since the AC links other brain regions in addition to the olfactory bulbs, it is likely involved in the functions of other brain regions. For instance, the amygdalae, also linked by the AC (Huang et al., 2019a), are known to control social behaviors, anxiety and fear memory and are relevant to ASD (Huang et al., 2014, 2019a). It is possible that the AC controls amygdalar activity to regulate amygdala-dependent behaviors. To explore this possibility, we applied electrophysiological recording and an optogenetic approach to first investigate the role of the AC in regulating amygdalar activity. The effects of AC transection on brain function were then evaluated by behavioral assays. Our results suggest that the AC regulates amygdalar activity and influences mouse behaviors, including locomotor activity, social interaction, and fear memory.

## Materials and Methods

### Animals

Male C57BL/6J mice at 6–8 weeks of age, purchased from the National Animal Research Laboratory (Taiwan), were used for all experiments in this report. Male mice were housed in the animal facility of the Institute of Molecular Biology, Academia Sinica, under controlled temperature and humidity and a 12 h light/12 h dark cycle with free access to water and chow (LabDiet #5010). All animal experiments were performed at 2–4 months of age with the approval (Protocol #14-11-759) of the Academia Sinica Institutional Animal Care and Utilization Committee and in strict accordance with its guidelines.

### Antibodies, Reagents, and Plasmids

The following antibodies and reagents were used in this study: C-FOS (9F6, rabbit, Cell Signaling, Beverly, MA); Neurobiotin^TM^ Tracer (SP-1120, Vector Laboratories); and 6-cyano-7-nitroquinoxaline-2,3-dione (CNQX, 0190, Tocris). The plasmids encoding adeno-associated virus (AAV) genome AAV-CaMKIIα-hChR2(C128S/D156A)-EYFP (Addgene plasmid #35501) were obtained from Dr. Karl Deisseroth (Lee et al., 2010; Yizhar et al., 2011).

### Stereotaxic Surgery and Optogenetic Stimulation

Mice were deeply anesthetized and placed on a Lab Standard™ Stereotaxic Instrument (Stoelting, Wood Dale, IL USA). After securing the animal to restrict its movement, 0.3 μl AAV solution (10^10^ vg/μl) was slowly infused over 10 min into the BLA (1.70 mm posterior, 3.3 mm lateral, and 4.5 mm ventral to the bregma), and then the optic fiber was implanted ipsilaterally into the same AAV injection site. For AC surgical lesioning, a home-made narrow razor blade (1.2 mm in width) was pushed into the brain at the bregma to a depth of 5 mm to cut the AC. Control (AC uncut) animals underwent the same procedure, except that the depth was changed to 4 mm so that the AC remained intact. This surgical procedure resulted in injury and bleeding of the superior sagittal sinus. However, owing to the fixation of the animals in the stereotaxic apparatus, the heart of the animal was located at a lower position than the brain, which slowed down the bleeding during surgery. Gentle press with cotton swab also stopped bleeding faster. Usually, bleeding stopped naturally within a minute. After surgery, mice acted normally in terms of eating and moving in their homecages. No subdural hematoma was found when the brains were removed from further analysis. We also confirmed that AC was completely disconnected in all AC cut mice but remained intact in all AC uncut mice. Some representative brains are shown in **Figures 3C,D**. For optogenetic stimulation and immunofluorescence staining, after a 2–3 weeks recovery from surgery, the mice were anesthetized by 2.5% isoflurane and stimulated with a 470 nm LED light (27 mW/mm^2^ at the tip of optical fiber) at 10 ms, 20 Hz for 20 s four times with 1-min intervals. Two hours after stimulation, mice were sacrificed, and subjected to immunostaining.

#### Slice Electrophysiology

**Figure 1:**
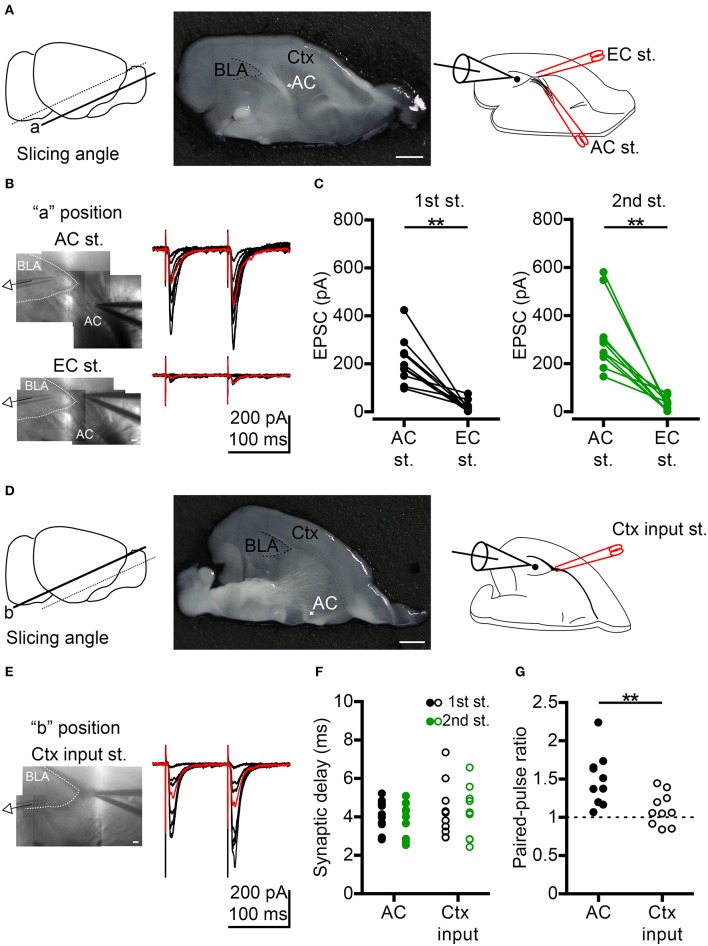
Comparison of anterior commissure (AC) and cortex (Ctx) inputs to BLA synaptic transmission. **(A)** Left, to preserve AC fibers, oblique horizontal slices were prepared by slicing at the angle indicated by the solid line (“a” position). Middle, photo shows the horizontal brain slice of the “a” position with the white AC fibers projecting to BLA (delineated by a dashed line). An example of half brain is shown. Right, schematic shows whole-cell recording of a BLA neuron during electrical stimulation at the AC followed by the external capsule (EC st.) outside the BLA. **(B)** Left, IR-DIC images show the voltage-clamp recording of a BLA neuron while stimulating AC (top) or EC (bottom) on the same slice shown in (**A**, middle). Right, example traces of EPSCs evoked by dual electrical stimulations of the AC (top) or EC (bottom) with the same series of stimulating intensities. Inter-pulse interval: 100 ms. Red traces represent 40–60% maximum AC-evoked EPSC (top) and EC-evoked EPSC (bottom) with the same stimulating intensity. **(C)** Comparison of the amplitudes of 40–60% maximum AC-evoked EPSC and EC-evoked EPSC in the 1st (left) and 2nd (right) stimuli. *n* = 10. **(D)** Left, horizontal brain slice prepared from a more dorsal oblique slicing plane (“b” position). Middle, photo shows the horizontal brain slice of the “b” position with no white AC fibers projecting to BLA (delineated by a dashed line). An example of half brain is shown. Right, schematic shows whole-cell recording of a BLA neuron during electrical stimulation of the Ctx inputs outside the BLA. **(E)** Left, IR-DIC image shows the voltage-clamp recording of a BLA neuron while stimulating Ctx inputs in the same slice shown in (**D**, middle). Right, example traces of EPSCs evoked by dual electrical stimulations of the Ctx inputs with a series of stimulating intensities. Inter-pulse interval: 100 ms. Red traces represent 40–60% maximum Ctx-evoked EPSC. **(F)** Synaptic delay of 40–60% maximum 1st EPSC (black) and 2nd EPSC (green) evoked by AC (dot) or Ctx inputs (open circle) stimulation. **(G)** Comparison of paired-pulse ratio evoked by paired stimuli of AC or Ctx inputs. In **(F,G)**, *n* = 10 for AC stimulation and *n* = 10 for significant Ctx inputs stimulation. AC, anterior commissure (arrow indicated); BLA, basolateral amygdala; Ctx, cortex. ^**^P < 0.01; Wilcoxon matched-pairs signed rank test was used for **(C)**, Mann-Whitney *U*-test was used for **(G)**. Scale bars: **(A,D)** middle, 1 mm; **(B,E)**, 100 μm.

Mice were anesthetized with isoflurane and transcardially perfused with ice-cold carbogenated (95% O_2_ and 5% CO_2_) sucrose solution (~30 mL) containing (in mM): 87 NaCl, 25 NaHCO_3_, 1.25 NaH_2_PO_4_, 2.5 KCl, 10 glucose, 75 sucrose, 0.5 CaCl_2_, and 7 MgCl_2_. Brains were dissected and sectioned in the same carbogenated sucrose solution using a vibrating tissue slicer (Microslicer^TM^ DTK-1000, Dosaka). Horizontal slices (300 μm thick) were prepared by slicing the brain through a series of oblique planes paralleling the hypothetical plane formed by connecting the ventral side of the olfactory bulb and the piriform cortex (Figure 1A) to better preserve AC axons. After incubating the slices at 34°C for 25 min and allowing recovery at room temperature (23 ± 2°C) in a holding chamber for at least 90 min, individual slices were then transferred to a submerged chamber for recording. The submerged chamber was continuously perfused with carbogenated artificial cerebrospinal fluid (ACSF) containing the following (in mM): 125 NaCl, 25 NaHCO_3_, 1.25 NaH_2_PO_4_, 2.5 KCl, 25 glucose, 2 CaCl_2_, and 1 MgCl_2_. Neurons were visually selected for recordings under an infrared differential interference contrast (IR-DIC) microscope (SliceScope, Scientifica) connected to a CCD camera (IR-1000, DAGE-MTI). Whole-cell and cell-attached recordings were performed with patch pipettes (4–8 MΩ) filled with an internal solution consisting of the following (in mM): 135.25 K-gluconate, 8.75 KCl, 0.2 EGTA, 4 MgATP, 10 HEPES, 7 Na_2_-phosphocreatine, 0.5 Na_3_GTP (pH 7.3 with KOH), and 0.3% Neurobiotin (wt/vol). For whole-cell recordings, pipette capacitance and series resistance were compensated (100% in current clamp and 70% in voltage clamp). For cell-attached recordings, giga-seal recordings were made in voltage-clamp mode with zero holding current. For AC electrical stimulation (200 μs), the slice containing AC fiber projections to BLA was used. The bipolar stainless steel stimulating electrodes (FHC) were placed on the AC fibers outside the BLA to avoid directly activating BLA circuits (Figures 1A,B). Cortex (Ctx) input stimulation (200 μs) was conducted on the same slice after the AC stimulation experiment. The bipolar stainless steel stimulating electrodes were placed on the putative external capsule (EC) near the AC fibers (Figures 1A,B). For significant Ctx input stimulation (200 μs), a horizontal slice taken from a more dorsal oblique plane lacking AC fiber extensions to BLA was used, and the bipolar stainless steel stimulating electrodes were placed on the EC (Figures 1D,E). For pharmacological experiments, input resistance (Rin) was monitored in each sweep. Data were discarded if Rin changed by >20% during the recording period. To distinguish projection neurons from interneurons, two firing properties were recorded in current-clamp mode: (1) the adapting firing pattern was recorded in response to step current injection, which elicits maximal spike number; and (2) fast afterhyperpolarization was recorded in response to rheobase current injection. Data were recorded with Multiclamp 700B amplifiers (Molecular Devices), filtered at 3 kHz, and sampled at 10 kHz with a Power 1401 mk II digitizer (Cambridge Electronic Design) controlled by Signal 4 software (Cambridge Electronic Design). The recording temperature was 23 ± 2°C. Data were analyzed offline using Clampfit 10.7 (Molecular Devices). Excitatory postsynaptic current (EPSC) and postsynaptic potential (PSP) amplitude (calculated from the pre-stimulation membrane potential to the peak of the PSP evoked by electrical stimulation) were analyzed for comparison of different input-evoked EPSCs, pair-pulse ratio measurement (2nd EPSC amplitude divided by 1st EPSC amplitude) and pharmacological experiments. Onset of the synaptic response was determined by the intersection of the baseline and a line through the 20 and 80% points of the rising phase of the first EPSC. Synaptic delay was calculated from the time of onset of electrical stimulation to the onset of the EPSC. For calculating spiking probability, 5–10 paired stimuli were delivered at each stimulus intensity. The plots of spiking probability against stimulus intensity were fitted with a sigmoid function *Y* = 100/[1 + 10∧*p*(*x*_0_ – *x*)] as described previously (Pouille et al., 2009), where *x*_0_ is the stimulus intensity at 50% spiking probability, and *p* is the slope at *x*_0_.

#### Reconstruction of the Cell Morphology of Neurobiotin-Filled Neurons

In order to confirm that the neurons recorded were projection neurons, the cells were loaded with Neurobiotin during recordings. The morphology of neurobiotin-filled neurons was visualized using immunofluorescence and cells with spiny dendrites were deemed projection neurons. To achieve this, brain slices were post-fixed after recording with 4% paraformaldehyde overnight at 4°C. After rinsing with phosphate buffered saline (PBS), permeabilization was conducted with 0.3% Triton X-100 in PBS (PBST). Neurobiotin signals were revealed by incubating slices with streptavidin-conjugated Alexa Fluor 647 (1:400; Invitrogen Inc.) in PBST overnight at 4°C. Labeled cells were imaged using a confocal laser scanner (LSM700, Carl Zeiss) equipped with a 20X/NA 0.8 DIC II objective lens (Plan-Apochromat, Carl Zeiss) and Zen 2009 (Carl Zeiss) acquisition and analysis software. Confocal image stacks were reconstructed with Neuromantic 1.6.3 (developed by Darren Myatt, University of Reading, UK). For detailed dendritic spine images, image stacks were acquired with the same confocal system but using a 63X/NA 1.4 oil objective lens (Plan-Apochromat, Carl Zeiss). To clearly present morphology and spiny dendrite signals, minimized adjustment of contrast and brightness were applied to the entire images and the Neurobiotin signals were inverted and transformed to grayscale images using Photoshop (Adobe).

#### Immunofluorescence Staining

Mice were perfused with 4% paraformaldehyde in PBS, and the brains were dissected and postfixed with 4% paraformaldehyde overnight at 4°C. Brains were cryopreserved in 30% sucrose at 4°C for 2 days and embedded in OCT (4583, Tissue-TeK). Fifty-μm-thick brain sections were cut with a cryostat microtome (CM1900, Leica). After rinsing with PBS, permeabilization was performed with 0.3% Triton X-100 in PBS (PBST) and blocked with 3% horse serum and 2% bovine serum albumin (BSA) in PBST at room temperature for 2 h. Primary antibody (anti-C-FOS, 1:200) was then added for overnight incubation at 4°C. After washing, sections were incubated with Alexa fluor 555-conjugated secondary antibodies (Invitrogen, Inc.). Counter stain using DAPI (4′,6-Diamidine-2′-phenylindole dihydrochloride) was included to label the nuclei of cells.

#### Image Acquisition, Processing, and Quantification

To show the anatomical position of the stimulating site and the location of the recorded neurons in slice electrophysiology, IR-DIC images were taken during recording using the setup for electrophysiological study described above. Multiple IR-DIC images of different locations were manually stitched together. After recording, the photos of the entire acute brain slices (post-fixed by 4% paraformaldehyde) were taken using a digital camera (EOS 5D mark III, Canon) coupled with a 100 mm lens (Canon EF 100 mm f/2.8 Macro USM). Mouse brain appearance was recorded using a standard cellphone camera. For hematoxylin and eosin (H&E) staining, true color images were acquired at room temperature under bright-field using a dissection microscope (MZ75, Leica) equipped with a digital camera (EOS 600D, Canon) to cover the entire brain area.

For C-FOS immunofluorescence staining, images were acquired using a confocal laser scanner (LSM700, Carl Zeiss) equipped with a 20X/NA 0.8 DIC II objective lens (Plan-Apochromat, Carl Zeiss) and Zen 2009 (Carl Zeiss) acquisition and analysis software. Several images were recorded at room temperature and assembled automatically to cover the entire BLA. To quantify C-FOS-positive cells in BLA, images were first imported into ImageJ (NIH). The BLA region was first outlined based on signals of external and internal capsules and DAPI (indicating the nuclei of cells). The area of BLA was also determined. Numbers of C-FOS-positive cells were then counted using the “Cell Count” plug-in. The density of C-FOS-positive cells could thus be determined by dividing the cell number by the area of BLA.

Images for publication were assembled using Photoshop. Minimized adjustments of contrast and brightness were applied to the entire images. To clearly present contralateral axons, the original monochrome image of ChR2-eYFP signal at the contralateral BLA was inverted and transformed to a grayscale image using Photoshop.

### Behavioral Analyses

Two to three-months-old male mice were randomly chosen for surgery. After surgery, mice were transferred to a behavioral room and single-housed for at least 14 days. Behavioral assays were carried out from 11:00 to 18:00, at least 2 h before the dark cycle. Mice were randomly selected for two sets of behavioral paradigms. The first set was open field and light-dark box, followed by elevated plus maze (Hung et al., 2018). The second set was reciprocal social interaction (RSI), followed by sucrose preference and auditory fear conditioning (AFC). Intervals between paradigms in the second set were about 7 days. In these sequential tasks, mice did not show any sign that prior tasks had elicited signs of stress or abnormal behavior. Behavioral analyses were performed blind.

#### Open Field

Mice individually explored an open box (transparent plastic box 40 × 40 × 30 cm) for 10 min. The behaviors of test mice were recorded from above by videotaping and then analyzed using the Smart Video Tracking System (Panlab) as described previously (Hung et al., 2018). The total moving distance indicates horizontal locomotory activity. The rearing number (counted manually) represents vertical locomotor activity and exploration. The time spent in the center area over the sum of time spent in both the center and corner areas (percentage) indicates anxiety of mice.

#### Light-Dark Box

An open black box (19 × 39 × 45 cm) was inverted and put into the open field box to divide it into two compartments, i.e., light box and black box, of equal size. A small opening (5 cm in diameter) in the side wall at the bottom of the black box allowed movement of mice between the two compartments. Mice were placed individually into the light compartment and then allowed to explore the apparatus for 10 min. Movement was recorded by videotaping and analyzed using the Smart Video Tracking System (Panlab, Barcelona, Spain). The time spent in the light box was measured to indicated the degree of anxiety.

#### Elevated Plus Maze

The maze consisted of two open-sided arms (30 × 5 cm) and two arms (30 × 5 cm) enclosed by a 14-cm-high wall. The central platform was a square of 5 cm and the entire apparatus was raised 45.5 cm above the floor. Mice were placed individually into the central area facing one of the open-sided arms and allowed to freely explore the maze for 10 min, as described previously (Chung et al., 2011; Lin and Hsueh, 2014). Their movements were recorded by video-recording from above and analyzed using the Smart Video Tracking System (Panlab, Barcelona, Spain). The percentage of time spent in the open-sided arms, the enclosed arms, and the central square was measured to evaluate the degree of anxiety of mice.

#### Reciprocal Social Interaction (RSI)

RSI was performed as described previously (Huang et al., 2014). During the test session, an unfamiliar wild-type untreated male mouse was put into the home cage of the isolated test mouse without replacing the lid of the cage. A patch of hair on the back of the unfamiliar mice was shaved beforehand to distinguish them from test mice. The social interaction of the test mouse with the unfamiliar mouse was recorded for 5 min using a digital camera. The time that the test mouse spent interacting with the unfamiliar mouse was measured to indicate RSI.

#### Sucrose Preference

The procedures for the sucrose preference test were adopted from the training procedure of the conditional taste aversion test described previously (Chung et al., 2011). Briefly, after pretraining mice to drink water in a training cage during a specific time window in the day for 7 days, the mice were presented with a sucrose-lithium chloride (LiCl) pairing, whereby they were first offered a sucrose solution (pleasant, new taste; 100 mM, 15 min) followed by an intraperitoneal injection of LiCl (malaise-inducing agent; 0.15 M, 20 μl/g of body weight). A diarrhetic response in mouse home cages was subsequently observed. As a control, NaCl instead of LiCl was injected into mice so that control animals did not experience malaise. Volumes of water drank on the last pretraining day and of sucrose solution on the training day were recorded to ensure normal water and sucrose uptake. Since AC cut mice did not exhibit a sucrose preference, the two-bottle test was not carried out to test their memory for taste aversion.

#### Auditory Fear Conditioning (AFC)

AFC was performed as previously described (Huang et al., 2014). During two pretraining days, mice were placed into a habituation chamber for 10 min. The average of freezing responses of these 2 days represented the “basal” freezing percentage (Basal). On the training day, mice were placed into a novel conditioning chamber for 4 min, followed by presentation of a 2-kHz, 80-dB, 18-s tone. Mice then immediately received a 0.6-mA, 2-s electric foot shock. The tone-shock pairings were performed three times with 1-min intervals. The freezing percentages within 1 min right after the last foot shock were also recorded to represent the response “after stimulation (AS),” indicating the sensation response to foot shock. On the next day, mice were placed in the habituation chamber for a 4-min period of free exploration, followed by exposure to twenty 20-s tones (2 kHz, 80 dB) delivered at 5-s intervals to assess auditory conditioned fear memory. The freezing response to auditory stimulation was measured. The average freezing percentage in response to the first four tones was taken as the degree of auditory fear conditioning (Memory). Freezing responses were recorded and measured with the FreezeScan^TM^ 2.0 system (CleverSys Inc.). To test whether increased neuronal activity ameliorated AC lesion defects, we intraperitoneally administered D-cycloserine into AC cut mice at a dose of 20 mg/kg. Thirty minutes after D-cycloserine treatment, mice were subjected to behavior tests.

### Statistical Analysis

Data are presented as means plus s.e.m. Data from individual animals are indicated as data points in the figures. Animals were randomly chosen for experiments. Behavioral analyses were conducted blind without knowing mouse genotype. No statistical method was applied to evaluate the sample size, but our sample sizes are similar to those of previous publications (Huang et al., 2014; Huang and Hsueh, 2017) and follow previously promoted principles (Charan and Kantharia, 2013). Our data meet test assumptions, e.g., normal distribution. Statistical comparisons were performed with unpaired Student's *t*-tests, Wilcoxon matched-pairs signed rank test, Mann-Whitney *U*-test using GraphPad Prism 5.0 (GraphPad Software) and two-way repeated measure ANOVA using SigmaStat 3.5 (Systat software) indicated in the figure legends. The detail statistical results are listed in Table S1.

## Results

### AC Input Activates BLA Neurons

To understand how the AC influences BLA, we first investigated synaptic transmission from the AC to BLA neurons by *ex vivo* slice electrophysiology. AC fibers were preserved in horizontal sections by slicing the brain at an oblique angle (Figure 1A, “a” position, left and middle panel). We applied electrical stimulation at the fiber tract of the posterior part of the AC outside BLA and performed whole-cell recordings on BLA neurons (Figure 1A, right panel). Bipolar electrode was placed on AC fibers outside the BLA to avoid direct stimulation of BLA microcircuits (Figure 1B, top). We found that excitatory postsynaptic currents (EPSCs) were evoked in recorded neurons (voltage-clamp recording, V_hold_ = −70 mV, near the inhibitory postsynaptic current reversal potential; [Cl^−^]_i_ = 8.75 mM) by dual electrical stimulation of the AC (inter-pulse interval, 100 ms) with different intensities (Figure 1B, top panel). To verify the specificity of AC electrical stimulation, bipolar electrodes were placed at the putative external capsule near AC fibers after examining the AC-evoked EPSCs, and we used the same series of stimulating intensities and paradigm (dual stimulation, inter-pulse interval, 100 ms) to evoke the synaptic transmission from external capsule to BLA. We found that it was very difficult to evoke EPSCs in BLA neurons under this condition (Figure 1B, bottom panel). Perhaps, the external capsule in the section of “a” position did not contain cortical afferents projecting to BLA. When we compared the synaptic responses evoked by AC stimulation vs. external capsule stimulation near-AC combined with the same stimulation intensity that yielded 40–60 % of maximal EPSC under AC stimulation, this external capsule-evoked EPSCs at “a” position were significantly smaller than those evoked by AC stimulation for both 1st and 2nd stimuli (Figure 1C). The amplitudes of the 1st and 2nd external capsule-evoked EPSCs were 15.5 ± 6.1% of 1st AC-evoked EPSCs and 12.9 ± 4.8% of 2nd AC-evoked EPSCs, respectively. Therefore, AC electrical stimulation is quite specific for our oblique horizontal slices and it is very unlikely that our AC stimulation evokes cortical afferents to cause BLA activation.

To record BLA response to the input from cortex (Ctx), we used more dorsal horizontal slices that lack AC fiber projections to BLA (“b” position). Indeed, significant Ctx-evoked EPSCs could be achieved (Figures 1D,E). We further compared these Ctx-evoked EPSCs with AC- evoked EPSCs (40–60% maximum) (Figure 1B AC vs. Figure 1E Ctx). No matter whether we assessed AC- or Ctx-evoked EPSCs, the mean synaptic delay of both the 1st EPSC and 2nd EPSC was shorter than 5 ms (Figure 1F), indicating that both AC-BLA and Ctx inputs-BLA synapses are monosynaptic connections. The short-term dynamics of AC-BLA synapses exhibited paired-pulse facilitation, whereas the Ctx inputs-BLA synapses showed less or no paired-pulse facilitation (Figure 1G), suggesting that the influence of the AC on BLA was greater during repetitive activities. This outcome further supports the specificity of our setup to analyze AC stimulation of BLA.

**Figure 2:**
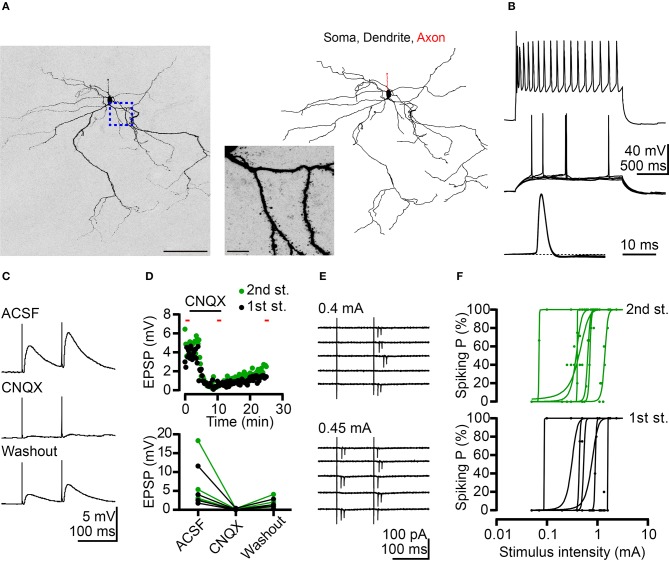
Potentiation of BLA neuronal activity by AC afferents. **(A)** A confocal Z-stack image of Neurobiotin signal (left) reveals neuronal morphology. Morphological reconstruction based on Neurobiotin signal is shown in the right panel. The middle panel is a high-magnification image corresponding to the region outlined by the blue dashed line. **(B)** Top, firing patterns of a putative projection neuron at maximal mean firing rate in response to current injection. Middle, superimposed traces showing the responses at threshold current injection. Bottom, horizontal enlargement of the first action potential from the middle row traces. Dashed line represents baseline of the action potential. **(C)** Example traces of EPSPs evoked by dual electrical stimulations of the AC recorded from a BLA neuron in current-clamp mode. Inter-pulse interval: 100 ms. Top: ACSF; middle: CNQX (10 μM) application; and bottom: washout of CNQX. **(D)** Top, time-course for the experiment shown in **(C)**. The duration of CNQX application was indicated. The time points of five sweeps in ACSF, CNQX application and washout conditions were indicated by red bars. The average traces of five sweeps in each condition were used to calculate the amplitude of EPSP for summary plots. Bottom, summary plots of the effect of CNQX on AC-BLA EPSPs. Black and green data-points represent the amplitude of the 1st EPSP and 2nd EPSP, respectively. *n* = 4. **(E)** Example traces indicate action currents recorded from a BLA neuron in cell-attached mode during dual electrical stimulations of the AC. Inter-pulse interval: 100 ms. Five sweeps are shown for each stimulation intensity (0.4 and 0.45 mA). **(F)** Plots of spiking probability (Spiking P) against stimulus intensity. Data are fitted with a sigmoid function. 1st stimulus induced spiking probabilities (black dots) and fitting curves (black lines) are shown in the bottom graph. Second stimulus induced spiking probabilities (green dots) and fitting curves (green lines) are shown in the top graph. *n* = 7. Scale bars, **(A)** left 100 μm; middle 10 μm.

The majority (~80%) of BLA neurons are projection neurons. In the absence of markers to distinguish cell types, we likely recorded projection neurons in our experiment. To confirm this point, Neurobiotin was loaded into neurons during recording to outline the cell morphology *post-hoc*. Using streptavidin to amplify the Neurobiotin signal, we could clearly reveal the characteristic spiny dendrites of recorded neurons (Figure 2A). The results of this experiment suggest that we were assessing projection neurons. Moreover, projection neurons can be distinguished from interneurons based on two electrophysiological features. First, we investigated the firing property of recorded neurons by incremental step current injections to show the adapting firing pattern (Figure 2B, top panel). The gradually reduced firing frequency that resulted from this approach is a feature of projection neurons. The other property is the small fast afterhyperpolarization (Figure 2B, middle and bottom panels). Based on these morphological and electrophysiological features, we ascertained that we recorded projection neurons in our experiments.

We also conducted current-clamp recordings to determine the net effect of AC to BLA activity. AC activation caused net depolarizing postsynaptic potentials (PSPs), which were blocked by glutamate receptor antagonist 6-cyano-7-nitroquinoxaline-2,3-dione (CNQX) (Figures 2C,D). Therefore, the glutamatergic synaptic transmission from the AC potentiates the activities of BLA projection neurons. Furthermore, in a subset of recordings, we examined the possibility that BLA projection neurons are recruited by AC activation. Recordings were made in the cell-attached mode to avoid interference with the intracellular milieu. Following cell-attached recordings, whole-cell recordings with Neurobiotin loading were also performed to confirm projection neuron identity as described above. For 7 out of 11 projection neurons, spikes (detected as extracellular action currents) were evoked by AC activation (Figures 2E,F). Stronger electrical stimulation of the AC increased spiking probability of BLA projection neurons (Figure 2E, top vs. bottom; Figure 2F). These results suggest that the AC can trigger action potentials in a subset (~60%) of BLA projection neurons.

Taken together, these results suggest that AC afferents form monosynaptic and glutamatergic connections with BLA projection neurons and deliver a positive signal to those neurons. Stronger AC input may trigger an action potential in BLA projection neurons.

### The AC Mediates the Connectivity Between Two Amygdalae

**Figure 3:**
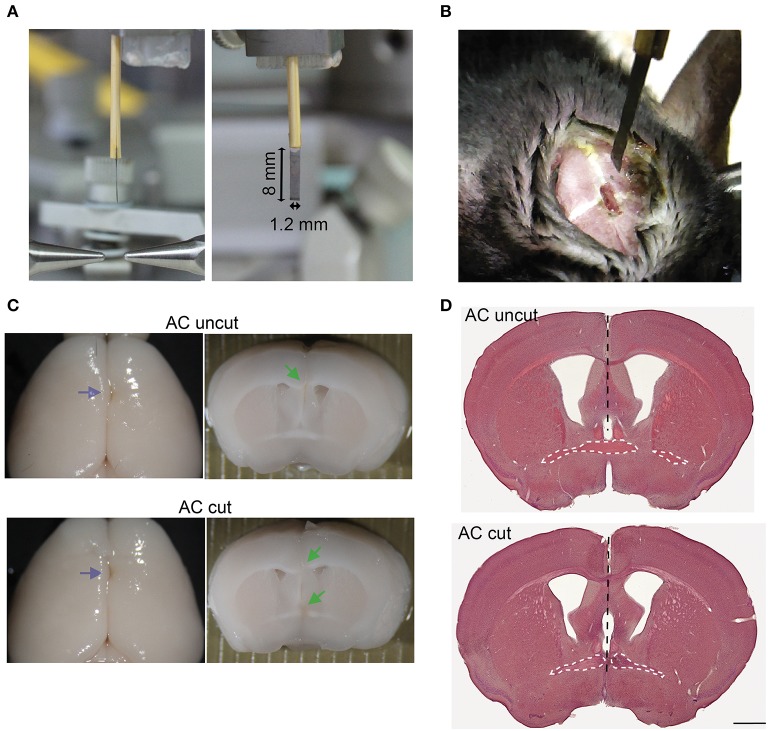
Surgical set-up for AC transection. **(A)** Photos of side and front views of a home-made narrow razor blade. The width and length of the blade are indicated. **(B)** An example of a mouse undergoing surgery. A small narrow hole was made at the bregma for the blade to penetrate. **(C)** Representative images of AC cut (bottom) and uncut (top) brains. Left, brain appearance after perfusion and post-fixation. Blue arrows point to blade insertion sites. Middle, coronal view of brains around the bregma. Green arrows indicate lesions of AC and CC. **(D)** HE staining of brain sections around the bregma. Black dashed lines indicate the path of the blade. The AC is also outlined by a white dashed line. Scale bar, 1 mm.

To further investigate the role of the AC in amygdalar activity *in vivo*, we conducted surgical lesioning using a narrow razor blade (Figures 3A,B, ~1.2 mm in width) to transect the AC at the midline (Figures 3B–D, AC cut). For a control (AC uncut), mice underwent the same surgical procedure but the blade did not penetrate deeply enough to cut the AC (Figures 3C,D). Thus, the major difference between AC cut and uncut mice is the lesioning of the AC. Other brain regions above the AC, including the CC and septum, were similarly lesioned in both AC cut and uncut mice.

**Figure 4:**
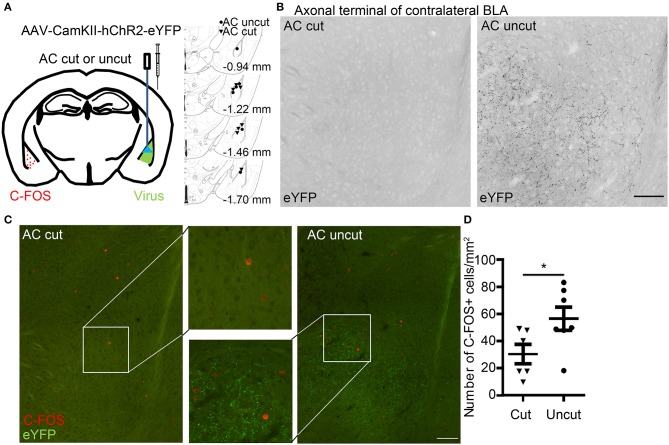
Optogenetic stimulation reveals contralateral connectivity of the basolateral amygdalae via the anterior commissure. **(A)** AAV-CaMKII-hChR2(C128S/D156A)-eYFP was unilaterally infected into the BLA of AC cut and AC uncut mice. Injection sites are indicated at right panel. Two weeks later, blue light stimulation was performed at the region with AAV infection. Two hours later, coronal sections of mouse brains were prepared for immunostaining. **(B)** BLA neurons expressing hChR2-eYFP extend their axon terminals to contralateral BLA in AC uncut mice but not AC cut mice. The inverted images of eYFP signals at contralateral BLA are shown in grayscale. **(C)** Neural activation at contralateral BLA relative to the light-stimulated site was monitored by C-FOS staining. ChR2-eYFP (green) labels axon terminals of BLA neurons. C-FOS signals are visualized in red. In **(B,C)**, the same images are shown, except that in **(B)** the images have been enlarged and processed into black/white using Photoshop to enhance the signal of ChR2-eYFP along axons. **(D)** Quantification of C-FOS^+^ cell number. Each data-point indicates the result of an individual mouse. Mean ± SEM are also shown. *P < 0.05; unpaired Student's *t*-test. Scale bars: **(B,C)**, 100 μm.

We then applied an optogenetic approach to investigate the role of the AC in amygdalar activity *in vivo*. Adeno-associated virus serotype 8 (AAV8) that expresses an hChR2-eYFP fusion protein under the control of the CaMKII promoter (AAV-CaMKII-hChR2(C128S/D156A)-eYFP) (Yizhar et al., 2011) was unilaterally infected into one BLA (Figure 4A). The AAV-infected site was labeled as the ipsilateral site. In AC uncut brains, we observed that ChR2-eYFP-positive axon terminals extended into contralateral BLA (Figure 4B, right). In AC cut mice, we did not find eYFP-positive axon terminals at contralateral BLA (Figure 4B, left), suggesting that amygdalar axon terminals labeled by ChR2-eYFP extended to the contralateral BLA via the AC (Figure 4B). When we used blue light to activate hChR2 at the AAV-injected side (Figure 4A), the population of cells with C-FOS immunoreactivity—an indicator of activated neurons—was increased at the contralateral BLA of AC uncut mice compared to AC cut mice (Figures 4C,D), suggesting that amygdalar neurons are able to innervate and activate the contralateral amygdala via the AC.

### AC Lesion Alters Locomotor Activities, Social Interaction, and Fear Memory

To further evaluate the behavioral significance of interamygdalar connections through the AC, we subjected AC cut and uncut mice to two sets of behavioral paradigms at least 2 weeks after surgery. The first set including open field, light-dark box, and elevated plus maze allowed us to analyze locomotor activity and anxiety. The second set comprising reciprocal social interaction, conditioned taste aversion and fear conditioning allowed us to evaluate social behaviors and amygdala-dependent associative memory. These paradigms require amygdalae and/or the olfactory system.

**Figure 5:**
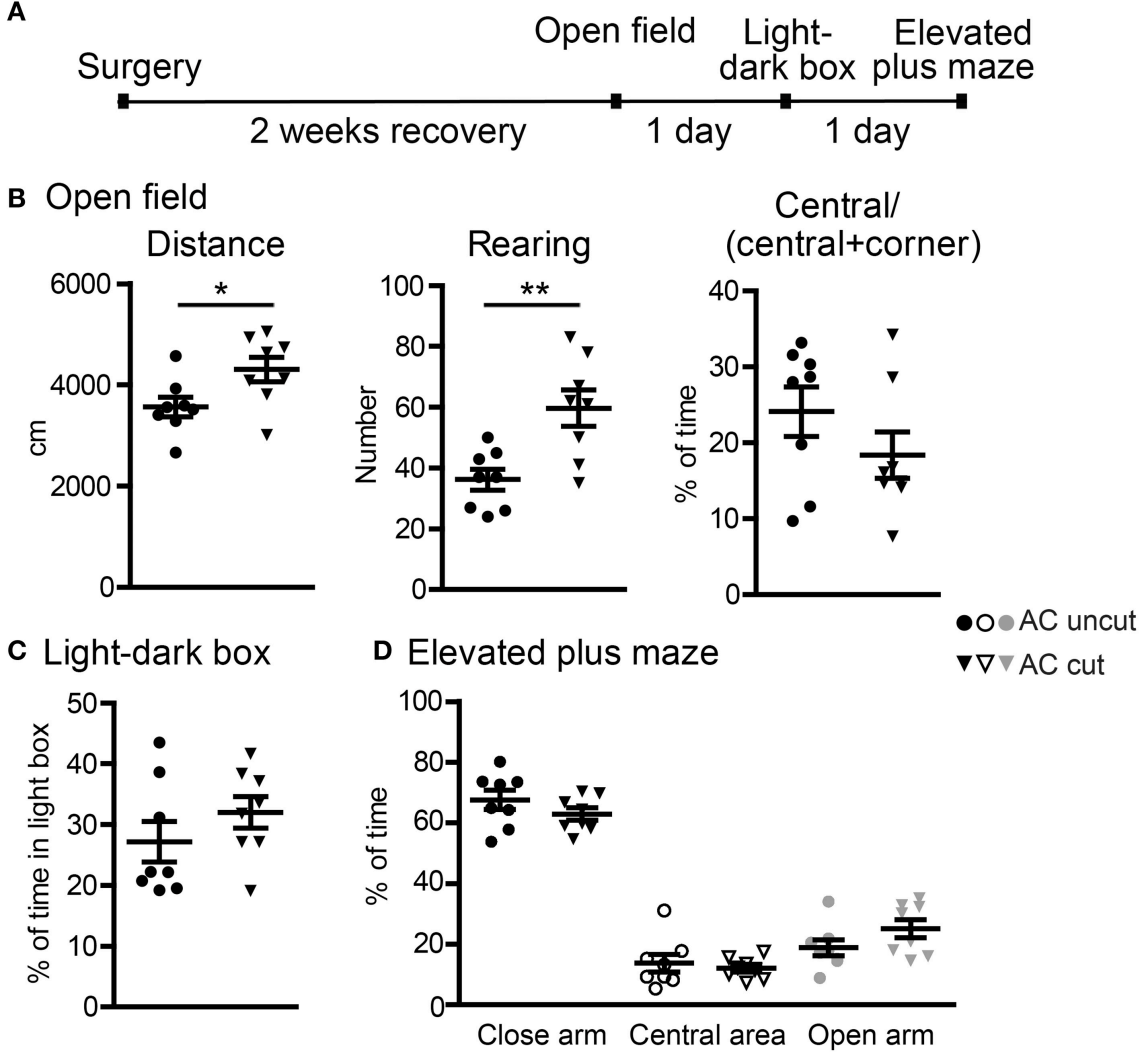
Surgical lesioning of the AC results in locomotor hyperactivity. **(A)** A flowchart of our behavioral paradigms. Both AC cut and AC uncut mice were used. **(B)** Open field. Total horizontal travel distance (left), vertical rearing number (middle), and the ratio of time spent at the central area to time spent at both central and corner regions. **(C)** Light-dark box. The percentages of time spent in the light box are shown. **(D)** Elevated plus maze. The percentages of time spent in the closed arm, central area and open arm are shown. Each data-point indicates the result of an individual mouse. Mean ± SEM are also shown. *P < 0.05; **P < 0.01. **(B**–**D)** unpaired Student's *t*-test.

In the first set of behavioral paradigms (Figure 5A), AC cut mice had a longer travel distance and a higher rearing activity in an open field compared with AC uncut mice (Figure 5B), suggesting locomotor hyperactivity caused by AC lesioning. Apart from hyperactivity, AC cut mice did not exhibit any other abnormality in open field, light-dark box, or elevated plus maze. Specifically, the tendency to stay in the center area of an open field, the light area of a light-dark box, and the open arm of an elevated plus maze was comparable between AC cut and uncut mice (Figures 5B–D). Together, these data suggest a role for the AC in controlling hyperactivity but not anxiety.

**Figure 6:**
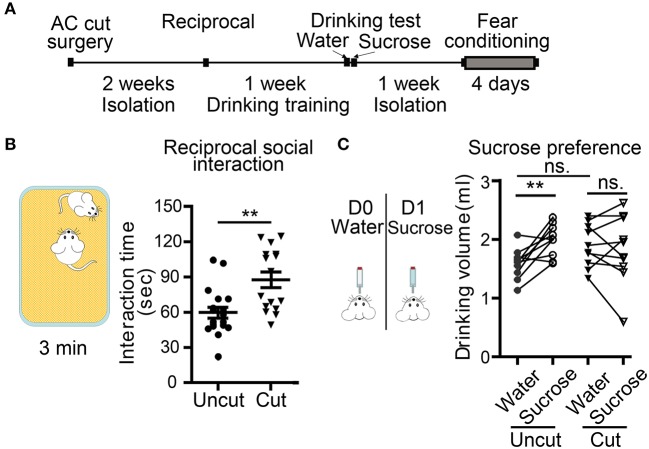
Surgical lesioning of the AC affects reciprocal social interaction and sucrose preference. **(A)** A flowchart of our behavioral paradigms. Both AC cut and AC uncut mice were used. **(B)** Reciprocal social interaction. Total interaction times for test mice to approach, chase and interact with the unfamiliar mouse are shown. **(C)** Sucrose preference. Comparison of the drinking volumes of water on day 0 (D0) and sucrose solution on day 1 (D1). Data for the same mouse are linked by a line. Each data point indicates the result of an individual mouse. Mean ± SEM are also shown. **P < 0.01, ns, not significant; **(B)** unpaired Student's *t*-test; **(C)** two-way repeated measure ANOVA.

In the second set of behavioral paradigms (Figure 6A), we found that compared with AC uncut mice, AC cut mice spent more time approaching and interacting with the unfamiliar male mice in reciprocal social interaction (Figure 6B). Some AC cut mice behaved more aggressively than AC uncut mice and even tried to mount unfamiliar male mice within the 5 min assay period (Movies S1, S2). These results suggest that AC transection results in altered social interaction.

Conditioned taste aversion is a robust assay for evaluating taste memory associated with nausea and/or vomiting responses (Lamprecht and Dudai, 1996; Lamprecht et al., 1997; Reilly and Bornovalova, 2005). Several brain regions, including amygdalae, are required for conditioned taste aversion (Lamprecht and Dudai, 1996; Lamprecht et al., 1997; Reilly and Bornovalova, 2005). Our AC uncut mice did exhibit a sucrose preference in our experiment (Figure 6C), but AC cut mice did not drink more sucrose solution than water (Figure 6C, AC cut mice). Since conditioned taste aversion relies on the display of a sucrose preference, loss of this sucrose preference made the conditioned taste aversion assay unsuitable for analyzing AC cut mice. Accordingly, we omitted the subsequent two-bottle test element of conditioned taste aversion in our mice, which aims to evaluate amygdala-dependent aversion memory.

**Figure 7:**
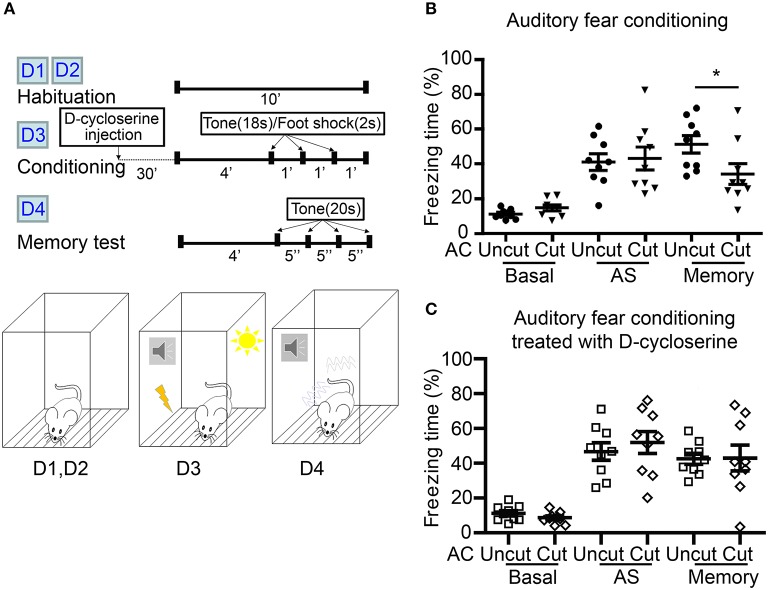
D-cycloserine mitigates fear memory deficits caused by AC transection. **(A)** Flowchart and schematic of auditory fear conditioning. The experiments were carried out over four consecutive days (D1–D4) as indicated. Both AC cut and AC uncut mice were subjected to the experiment. Half of the mice were intraperitoneally injected with D-cycloserine 30 min before conditioning at D3. **(B)** Freezing percentage of both AC uncut and AC cut mice in the absence of D-cycloserine treatment. **(C)** D-cycloserine treatment ameliorates the defects of AC cut mice in auditory fear memory. Basal, the freezing response during habituation; AS, after stimulation; Memory, the average freezing percentage in response to the first four tones at day 4. Each data point indicates the result of an individual mouse. Mean ± SEM are also shown. *P < 0.05; unpaired Student's *t*-test for **(B,C)**.

We then used auditory fear conditioning—a paradigm highly relevant to amygdalae but irrelevant to olfaction—to analyze AC cut mice (Figure 7A). Compared with AC uncut mice, AC cut mice behaved normally during habituation (labeled as “basal” in Figure 7B) and immediately after foot shock stimulation (labeled as “AS” to represent directly after stimulation in Figure 7B). This outcome suggests that AC cut mice behaved normally with regard to pain sensation and immediate fear response to foot shock. However, 1 day after training, AC cut mice had a lower freezing response when tested for fear memory (Figure 7B), supporting that AC lesion impairs auditory fear memory, which is a behavior requiring functional amygdalae.

Our previous study showed that increased NMDAR activity by both local infusion of D-cycloserine to the BLA and systemic administration of D-cycloserine and clioquinol ameliorate a deficit in auditory fear memory of *Tbr1*^+/−^ mice, in which the posterior part of the AC is deficient (Huang et al., 2014; Lee et al., 2015). Recently, we further demonstrated that contralateral BLA stimulation promotes synaptic facilitation of BLA projection neurons in a NMDAR-dependent manner (Huang et al., 2019a). We then wondered if D-cycloserine treatment could also improve the fear memory of AC cut mice. To assess this possibility, we systematically treated AC cut and uncut mice with D-cycloserine 30 min before training and found that both types of mice had similar freezing responses directly after foot shock stimulation (Figure 7C, AS). Importantly, the freezing responses of AC cut mice in the auditory fear memory test were also comparable to those of AC uncut mice in the presence of D-cycloserine (Figure 7C, memory), suggesting that increased NMDAR activity ameliorates the behavioral defects caused by AC lesioning.

## Discussion

In this report, we employed various approaches including electrophysiological recording, optogenetic stimulation and behavioral analyses to investigate the role of the AC in the brain functions of mice. *ex vivo* slice recording at BLA was applied to demonstrate that contralateral afferents via the AC form monosynaptic connections with BLA projection neurons. The synaptic contacts are glutamatergic and exhibit facilitating short-term dynamics. Thus, contralateral afferents via AC provide a positive signal to potentiate BLA neurons. Since the AC contains axonal projections derived from several different brain regions, it remains unclear if there is a specific input from a particular brain region that activates BLA neurons or if multiple regions exert similar actions to activate BLA neurons. Nevertheless, our *in vivo* study using optogenetic stimulation demonstrates that activation at one side of BLA can increase neuronal activity (as indicated by C-FOS expression) of the other BLA. Consistent with this point, our recent study also indicated that the two BLAs in the two hemispheres potentiate synaptic responses of each other (Huang et al., 2019a). In addition to BLA, it will be interesting to further analyze the function of contralateral projections derived from other brain regions, which should help reveal how contralateral afferents via the AC control brain activity and function.

In our AC transection experiment, a narrow blade (~1.2 mm in width) was pushed into the brain at the bregma. Brain regions above the AC, including the CC and septum, were also lesioned by transection. Thus, surgical control (AC uncut) mice that went through the same procedure were always included for comparison. Since the AC remains intact in AC uncut mice and the brain regions above the AC are lesioned in both AC uncut and cut mice, our findings evidence the significance of the AC in the behaviors we examined. However, given that locomotor activity, social interaction and fear memory are all complex behaviors involving multiple brain regions, lesioned brain areas above the AC may also contribute to abnormal behaviors, but we assert that AC lesioning is definitely crucial to those behavioral deficits. More detailed circuit analyses using other approaches will help address if lesioning of the regions above the AC contribute to the functioning of the AC and BLA and if it can influence mouse behaviors.

We here use ChR2-eYFP to visualize contralateral axonal projection derived from the region containing BLA. ChR2-eYFP was originally designed for optogenetic stimulation to investigate neural circuits (Boyden et al., 2005). It can be distributed throughout entire neurons, including the soma and axons (Gradinaru et al., 2007; Huang et al., 2019a). Thus, light stimulation can be applied at both soma and axonal terminals to activate ChR2-eYFP (Gradinaru et al., 2007; Huang et al., 2019a). Based on this property, we used ChR2-eYFP here for two purposes, i.e., optogenetic activation and to serve as an axonal marker to visualize contralateral projections.

In addition to the AC, other interhemispheric connectivity has also been investigated. Somatosensory and motor cortices in the two brain hemispheres also innervate each other via the CC. The transcallosal axons tend to activate inhibitory neurons and consequently deliver an inhibitory signal to contralateral cortices (Meyer et al., 1995; Palmer et al., 2012). For the hippocampal commissure, commissural axons derived from hilar mossy cells also target interneurons to suppress granule cell spiking at the contralateral dentate gyrus (Buzsaki and Eidelberg, 1981; Hsu et al., 2016). Thus, in cortex and hippocampus, interhemispheric connectivity delivers an inhibitory signal between the two hemispheres, which differs from the potentiation effect of contralateral AC afferents on BLA. Thus, the effects of different contralateral projections vary.

Since the AC mediates contralateral connections between olfactory bulbs and amygdalae, we anticipated AC cut mice would exhibit abnormalities in olfaction and amygdala-dependent behaviors if interhemispheric connection is indeed critical for the functions of those two brain regions. A previous study demonstrated that contralateral connectivity between two olfactoray bulbs is crucial for odor source localization (Esquivelzeta Rabell et al., 2017). Consistent with that finding, our AC cut mice did not exhibit a sucrose preference, most likely due to olfactory impairment. With regard to social interaction, our current study shows that male AC cut mice became more active in approaching an unfamiliar mouse and even displayed mounting behavior toward other male mice. However, our previous study showed that *Tbr1*^+/−^ mice characterized by hypoplasia/absence of the posterior part of the AC exhibited reduced social interaction (Huang et al., 2014, 2019a). Four possibilities may account for this difference and these four possibilities are not excluded from each other. First, contralateral connectivity via both the anterior and posterior parts of the AC were completely disrupted by AC transection. However, the anterior part of the AC is still present in *Tbr1*^+/−^ mice, though it is significantly smaller. Thus, some olfactory functions of *Tbr1*^+/−^ mice are retained, such as olfactory sensation, but olfactory discrimination is impaired (Huang et al., 2019b). Second, the brain regions above the AC were also lesioned in our AC cut mice. Those regions may also modulate social behaviors, so lesioning of them may result in different behavioral outcomes. Third, the AC connects olfactory bulbs, amygdalae as well as other ventral regions of cerebral cortex (including the olfactory tubercles), the anterior piriform cortices, the perirhinal cortices, and the entorhinal cortices (Horel and Stelzner, 1981; Jouandet and Hartenstein, 1983). Thus, the links between these cortical regions were also completely severed by AC transection. These additional defects may have also influenced the outcome of our behavioral analyses. Finally, *Tbr1* deficiency is a developmental defect, which is likely to be influenced by other compensatory effects. In contrast, AC transection is an acute way of disconnecting all established connections via the AC in adult mice. Although the results of our AC transection experiments support the role of the AC in social interaction, echoing the involvement of disrupted interhemispheric connectivity in ASD, this acute effect actually differs from a developmental effect, perhaps due to different timing. It would be intriguing to further dissect why AC transection has a contrasting effect on social behaviors relative to that shown by *Tbr1*^+/−^ mice.

In addition to altering social interaction and olfaction, AC transection impaired auditory fear memory and we show that systemic administration of D-cycloserine, an NMDAR co-agonist, ameliorates the memory deficit caused by AC transection. Therefore, enhancement of the ipsilateral signals by D-cycloserine seems sufficient to ameliorate the deficits caused by AC transection, suggesting that the function of contralateral afferents is to intensify ipsilateral signals. This supposition is consistent with our recent finding that contralateral BLA stimulation promotes synaptic facilitation of BLA projection neurons (Huang et al., 2019a). This kind of BLA contralateral enhancement requires NMDAR because AP-5, an antagonist of NMDAR, attenuates the synaptic facilitation induced by contralateral BLA (Huang et al., 2019a). Our electrophysiological recordings also indicate that contralateral afferents via the AC form glutamatergic synapses with BLA projection neurons and activate them. Together, these findings suggest that interhemispheric connectivity via the AC is critical to providing the positive signal mediated by glutamatergic synapses and thereby influencing the function of BLA in fear memory.

Apart from the evidence from *Tbr1*^+/−^ mice, a recent study on ASD patients also indicated that hypoplasia/absence of the AC is a common feature of the brain anatomy of patients carrying mutations in the *TBR1* gene (Nambot et al., 2020). That finding suggests that AC deficits are an evolutionarily conserved feature of TBR1 deficiency in rodents and human and strengthens the evidence that defective AC development is important for ASD-related phenotypes. However, apart from their impact on the AC, *Tbr1* mutations have been shown to influence the expression of NMDAR and other proteins (Wang et al., 2004a,b; Chuang et al., 2014; Huang et al., 2014). Accordingly, other deficits caused by *Tbr1* mutation may also be involved in ASD-related phenotypes. In the current report, we performed surgical lesioning of adult mice to show that disruption of the AC alters social interaction in rodents. Our findings further evidence the involvement of connectivity between the two brain hemispheres via the AC for regulating social interaction. We advocate using structural magnetic resonance imaging (MRI) to investigate AC deficits in ASD patients to further explore this topic.

## Conclusion

Our study provides evidence that disruption of the AC alters BLA neuronal activity, locomotor activity, social interaction and fear memory in mice. Both *ex vivo* recording and *in vivo* optogenetic stimulation suggest that contralateral afferents via the AC activate BLA neurons. Behavioral assays also support that amygdalar function is impaired in AC-transected mice because auditory fear memory and social interaction are altered by AC transection. Since the AC also links the two anterior piriform cortices, the perirhinal cortices and the entorhinal cortices in rodents, it will be intriguing to further dissect the impact of the AC on neuronal responses of these cortical regions and on behaviors related to these regions. Such investigations will shed light on the role of interhemispheric connectivity in brain activity and function.

## Data Availability Statement

All datasets generated for this study are included in the article/Supplementary Material.

## Ethics Statement

All animal experiments were performed with the approval (Protocol #14-11-759) of the Academia Sinica Institutional Animal Care and Utilization Committee and in strict accordance with its guidelines.

## Author Contributions

T-TH and T-NH designed, performed, analyzed experiments, and wrote the manuscript. Y-PH performed the project planning, experimental design, manuscript writing, and secured funding support. All authors approve the final version for publication.

### Conflict of Interest

The authors declare that the research was conducted in the absence of any commercial or financial relationships that could be construed as a potential conflict of interest.

## Supplementary Material

Movies S1, S2 Two sets of reciprocal social interaction.
