# Supplementary material for: Anterior Commissure Regulates Neuronal Activity of Amygdalae and Influences Locomotor Activity, Social Interaction and Fear Memory in Mice
Source: Front Mol Neurosci. 2020 Mar 31;13:47. doi: 10.3389/fnmol.2020.00047 (PMC7136557; doi:10.3389/fnmol.2020.00047)
Supplement: Supplementary file 1 [file Table_1.pdf]

Table 1. Statistical Results														
Figure		Assay Performed	Parameter (Unit)	Groups	N	Descriptive Statistics	Statistical Analysis					Column statistics		
						Average ± SEM	Statistical Test			Significance		Factor	Comparison	P value
1	C-1	EPSC amplitude comparison	EPSC amplitude (pA)				Statistical Test		P value					
				1st. AC st	10	211.3 ± 30.58	Wilcoxon matched-pairs signed rank		0.002		Two-tail			
				1st Ctx st	10	22.61 ± 7.548								
	C-2		EPSC amplitude (pA)				Statistical Test		P value					
				2nd AC st	10	308.6 ± 45.97	Wilcoxon matched-pairs signed rank		0.002		Two-tail			
				2nd Ctx st	10	29.69 ± 9.084								
1	G	Paired-pulse ratio comparison	Paired-pulse ratio				Statistical Test							
				AC	10	1.496 ± 0.1088	Mann Whitney U test		P value		Two-tail			
				Ctx input	10	1.108 ±			0.0068					
4	D	IHC (c-FOS+ cells in BLA)	Number of c-FOS+ cell / mm^2				Statistical Test		P value					
				AC cut	6	30.49 ± 7.053	Unpaired T test		0.0405	t(11)=2.321	Two-tail			
				AC uncut	6	56.48 ± 8.401								
5	B-1	Open field-distance	cm				Statistical Test		P value					
				AC uncut	8	3567 ± 192.3	Unpaired T test		0.0309	t(14)=2.4	Two-tail			
				AC cut	8	43.6 ± 240.7								
	B-2	Open field-rearing	Number				Statistical Test		P value					
				AC uncut	8	36.13 ± 3.414	Unpaired T test		0.0041	t(14)=3.42	Two-tail			
				AC cut	8	59.63 ± 5.964								
	B-3	Open field-cental/corner rate	% of time				Statistical Test		P value					
				AC uncut	8	24.13 ± 3.263	Unpaired T test		0.2204	t(14)=1.283	Two-tail			
				AC cut	8	18.4 ± 3.055								
5	C	Light-dark box	% of time in light box				Statistical Test		P value					
				AC uncut	8	27.14 ± 3.349	Unpaired T test		0.2704	t(14)=1.148	Two-tail			
				AC cut	8	32 ± 2.594								
5	D-1	Elelavated plus maze - close arm	% of time				Statistical Test		P value					
				AC uncut	8	67.57 ± 3.154	Unpaired T test		0.2347	t(14)=1.242	Two-tail			
				AC cut	8	62.9 ± 2.048								
	D-2	Elelavated plus maze - central area	% of time				Statistical Test		P value					
				AC uncut	8	13.68 ± 2.861	Unpaired T test		0.6181	t(14)=0.5099	Two-tail			
				AC cut	8	12.09 ± 1.223								
	D-3	Elelavated plus maze - open arm	% of time				Statistical Test		P value					
				AC uncut	8	18.76 ± 2.6	Unpaired T test		0.136	t(14)=1.582	Two-tail			
				AC cut	8	25.02 ± 2.981								
6	B	Reciprocal social interacation	Interaction time				Statistical Test		P value					
				AC uncut	19	59.81 ± 4.527	Unpaired T test		0.0012	t(33)=3.546	Two-tail			
				AC cut	16	87.57 ± 6.629								
6	C	Sucrose preference	Drinkingvolume(ml)				2-way RM ANOVA					Multiple Comparison Procedures (Bonferroni t-test)		P value
				AC uncut- Water	10	1.592 ±	Surgury:: F(1,18)=0.203, P=0.657<br>Drink::F(1,18)=4.385, P=0.051<br>Interaction:: F(1,18)=8.026, P=0.011					Col 1	AC uncut VS. AC cut	0.657
				AC uncut-	10	1.992 ± 0.08512						Col 2	Water VS. Sucrose	0.051
				AC cut-Water	10	1.892 ± 0.115						Col 2 with AC uncut	Water VS.Sucrose	0.003
				AC cut-Sucrose	10	1.832 ± 0.1852						Col 2 with AC cut	Water VS.Sucrose	0.608
												Col 1 with Water	AC uncut VS. AC cut	0.098
												Col 1 with Sucrose	AC uncut VS. AC cut	0.369
7	B-1	Auditory fear conditioning - Basal	% of freezing time				Statistical Test		P value					
				AC uncut	9	11.29 ± 0.8988	Unpaired T test		0.0729	t(16)=1.92	Two-tail			
				AC cut	9	14.88 ± 16.40								
	B-2	Auditory fear conditioning - AS	% of freezing time				Statistical Test		P value					
				AC uncut	9	48.7 ± 5.301	Unpaired T test		0.9421	t(16)=0.07377	Two-tail			
				AC cut	9	48.09 ± 6.494								
	B-3	Auditory fear conditioning - Memory	% of freezing time				Statistical Test		P value					
				AC uncut	9	53.61 ± 3.993	Unpaired T test		0.012	t(16)=2.832	Two-tail			
				AC cut	9	34.43 ± 5.474								
7	C-1	Auditory fear conditioning with D-cycoserine- Basal	% of freezing time				Statistical Test		P value					
				AC uncut	9	11.21 ± 1.507	Unpaired T test		0.1928	t(16)=1.36	Two-tail			
				AC cut	9	8.656 ± 1.116								
	C-2	Auditory fear conditioning with D-cycoserine- AS	% of freezing time				Statistical Test		P value					
				AC uncut	9	46.75 ± 4.973	Unpaired T test		0.5277	t(16)=0.6456	Two-tail			
				AC cut	9	51.94 ± 6.322								
	C-3	Auditory fear conditioning with D-cycoserine- Memory	% of freezing time				Statistical Test		P value					
				AC uncut	9	42.61 ± 3.122	Unpaired T test		0.9592	t(16)=0.05193	Two-tail			
				AC cut	9	43.03 ± 7.392								
